# GSK3β inhibition restores cortical gamma oscillation and cognitive behavior in a mouse model of NMDA receptor hypofunction relevant to schizophrenia

**DOI:** 10.1038/s41386-020-00819-0

**Published:** 2020-08-28

**Authors:** Kazuhito Nakao, Mahendra Singh, Kiran Sapkota, Bailey C. Hagler, Robert N. Hunter, Chander Raman, John J. Hablitz, Kazu Nakazawa

**Affiliations:** 1 Department of Neuroscience, Southern Research, Birmingham, AL 35205 USA; 2 Department of Psychiatry and Behavioral Neurobiology, University of Alabama at Birmingham, Birmingham, AL 35294 USA; 3 Department of Chemistry, Southern Research, Birmingham, AL 35205 USA; 4 Department of Medicine, University of Alabama at Birmingham, Birmingham, AL 35294 USA; 5 Department of Neurobiology, University of Alabama at Birmingham, Birmingham, AL 35294 USA

**Keywords:** Schizophrenia, Neurophysiology, Cognitive control

## Abstract

Cortical gamma oscillations are believed to be involved in mental processes which are disturbed in schizophrenia. For example, the magnitudes of sensory-evoked oscillations, as measured by auditory steady-state responses (ASSRs) at 40 Hz, are robustly diminished, whereas the baseline gamma power is enhanced in schizophrenia. Such dual gamma oscillation abnormalities are also present in a mouse model of *N*-methyl-D-aspartate receptor hypofunction (Ppp1r2cre/*Grin1* knockout mice). However, it is unclear whether the abnormal gamma oscillations are associated with dysfunction in schizophrenia. We found that glycogen synthase kinase-3 (GSK3) is overactivated in corticolimbic parvalbumin-positive GABAergic interneurons in *Grin1* mutant mice. Here we addressed whether GSK3β inhibition reverses both abnormal gamma oscillations and behavioral deficits with high correlation by pharmacological and genetic approach. We demonstrated that the paralog selective-GSK3β inhibitor, but not GSK3α inhibitor, normalizes the diminished ASSRs, excessive baseline gamma power, and deficits in spatial working memory and prepulse inhibition (PPI) of acoustic startle in *Grin1* mutant mice. Cell-type specific *GSK3B* knockdown, but not *GSK3A* knockdown, also reversed abnormal gamma oscillations and behavioral deficits. Moreover, *GSK3B* knockdown, but not *GSK3A* knockdown, reverses the mutants’ in vivo spike synchrony deficits. Finally, ex vivo patch-clamp recording from pairs of neighboring cortical pyramidal neurons showed a reduction of synchronous spontaneous inhibitory-postsynaptic-current events in mutants, which was reversed by GSK3β inhibition genetically and pharmacologically. Together, GSK3β inhibition in corticolimbic interneurons ameliorates the deficits in spatial working memory and PPI, presumably by restoration of synchronous GABA release, synchronous spike firing, and evoked-gamma power increase with lowered baseline power.

## Introduction

The disturbance of neural oscillations in the gamma frequency band (30–100 Hz) is considered to be a core pathophysiological feature of schizophrenia, particularly in the generation of cognitive dysfunction [1, 2]. For example, clinical electroencephalogram has shown reduction of auditory steady-state responses (ASSRs) at 40 Hz in patients with schizophrenia, which are electrophysiological responses entrained to the frequency and phase of a periodic auditory stimulus generated by auditory cortex activity [3, 4]. The impairment of visual-evoked gamma oscillation in schizophrenia was also observed during Gestalt stimuli [5]. In addition to impairment in evoked gamma oscillations, spontaneous or baseline gamma-band cortical activity during the resting state is also reported in schizophrenia [6–8]. However, it is unclear whether such abnormal gamma oscillations are causally linked to cognitive impairments in schizophrenia.

Preclinically, administration of uncompetitive *N*-methyl-D-aspartate receptor (NMDAR) antagonists, such as ketamine and MK-801, in rodents dose-dependently reduces sensory-evoked gamma responses [9, 10] and increases spontaneous or baseline gamma oscillations [11–13]. Furthermore, positive associations have been reported between abnormalities in EEG gamma power and deficits in prepulse inhibition (PPI) of acoustic startle induced by the NMDAR antagonists, suggesting that gamma power abnormalities may be responsible for sensory processing disturbance [14]. Schizophrenia-related behaviors, including PPI and Y-maze alternation (to assess spatial working memory), are also defective in a genetically-engineered mouse model of NMDAR hypofunction [15]. In this model, indispensable NMDAR subunit *Grin1* is missing from ~50% of cortical and hippocampal GABAergic neurons, a majority of which are parvalbumin (PV)-positive fast-spiking interneurons, in early postnatal development (Ppp1r2cre/*Grin1* knockout (KO) mice) [16]. Notably, 40-Hz click train-evoked ASSR deficits and spontaneous local field potential broad-band power increase in the pre-stimulus period are both observed in this mutants [17]. Genetic *Grin1* deletion selectively from PV neurons in awake mice also resulted in increased baseline gamma power [18, 19] and impaired optogenetically evoked-gamma oscillations [19], suggesting that *Grin1*-deleted PV neurons play a role in abnormal gamma oscillations. However, it remains to be addressed whether abnormal gamma oscillations are strongly associated with cognitive dysfunction in the genetic NMDAR hypofunction model.

Glycogen synthase kinase-3 (GSK3) is a Ser/Thr protein kinase that is ubiquitously expressed in all mammalian tissues and subcellular organelles, including the brain with high expression. Accumulating evidence suggests that GSK3 regulatory pathways are altered and GSK3 activity increases in the schizophrenia [20, 21]. GSK3 comprises two structurally and functionally related serine/threonine kinases encoded by two distinct genes, *GSK3A* and *GSK3B*. Both kinases are inherently active under resting conditions, and are primarily regulated by phosphorylation at two levels: (i) inhibitory phosphorylation of serine residues S21/S9 in GSK3α/β and (ii) tyrosine phosphorylation at Y279/Y216 in GSK3α/β, which augments their activity and relieves substrate-priming by other kinases [22]. Notably, nonselective GSK3 inhibitors have been shown to alleviate the behavioral impairments induced by ketamine treatment [23] and in the 22q11.2 microdeletion mouse model [24].

Here we report that proper GSK3β activity in corticolimbic GABAergic neurons is crucial for the emergence of the gamma oscillation and cognitive function in aforementioned *Grin1* mouse model of NMDAR hypofunction [16]. To determine which isoform of GSK3, GSK3α vs GSK3β, is critically involved in the gamma oscillations, we used the paralog-selective GSK3 inhibitors, BRD0705 for GSK3α and BRD3731 for GSK3β. To further determine whether GSK3 in corticolimbic GABAergic neurons is crucial for the emergence of abnormal gamma oscillations and behaviors, we have generated a floxed-*GSK3A* mouse line to conditionally delete the *GSK3A*, with the complimentary use of a floxed-*GSK3B* mouse strain. We assess whether both deficits in ASSRs and cognitive behavior could consistently be reversed by manipulation of GSK3β activity in the *Grin1* mutant mice.

## Materials and methods

All experimental procedures were approved by the Institutional Animal Care and Use Committee at University of Alabama at Birmingham and Southern Research. For detailed experiential procedures, see Supplementary Information.

### Animals

We employed *Ppp1r2*-cre/floxed-*Grin1* KO mice (or simply *Grin1* mutant mice), in which genetic deletion of obligatory *Grin1* subunit is introduced in ~50% cortical and hippocampal GABAergic interneurons from the postnatal second week [16]. To reduce the kinase activity of GSK3A or GSK3B in *Grin1*-deleted GABAergic neurons, either floxed-GSK3A or floxed-GSK3B mouse strain was bred to *Grin1* mutant mice to generate GABAergic neuron-specific GSK3 heterozygous KO mice (namely, GSK3A- or GSK3B-knockdown mice). The generation of floxed-GSK3A mouse strain is described in Supplementary Fig. S1. The floxed-GSK3B strain was obtained from Dr. J. Woodgett [25].

### Immunohistochemistry

Immunohistochemistry was performed as previously described [16]. Briefly, sections containing auditory cortex were double-immunostained with rabbit anti-GSK3α (1:200, G08-63R-25, SignalChem, USA), rabbit anti-GSK3β (1:1000, ab32391, Abcam, USA), or rabbit GSK3β phospho-Y216 (1:150, ab75745 Abcam, USA), and mouse anti-PV (1:5000, 235, Swant, Switzerland). Images of the auditory cortex were captured using confocal microscope (Nikon A1). NIH ImageJ software was used to measure the integrated density of GSK3α, GSK3β and GSK3-phospho (Y279/Y216) in PV-positive interneurons or non-PV neurons. The corrected total cell fluorescence (CTCF) was calculated as previously described [26]. Results are presented as normalized florescence, which is the ratio of CTCF value of the protein to the CTCF values of corresponding proteins in non-PV neurons of floxed-control mice.

### GSK inhibitors

A non-selective GSK3 inhibitor, SB216763 (2.5 mg/kg, *i.p*.; Tocris, Ellisiville, MO) was dissolved in 10% DMSO and 10% Tween80/PBS. Another non-selective GSK3 inhibitor, TDZD-8 (2.5 mg/kg, *i.p*; Abcam, Cambridge, MA) was dissolved in 1.25% DMSO and 5% Tween80/PBS. These two non-selective GSK3 inhibitors are brain-permeable as previously described [27, 28]. The paralog-selective GSK3β inhibitor BRD3731 (30 mg/kg, i.p.) and paralog-selective GSK3α inhibitor BRD0705 (30 mg/kg, i.p.) were synthesized by referring to the protocols [29]. BRD3731 and BRD0705 were dissolved in 2% DMSO and 2% Tween80/PBS. Each was administered to the mice one hr before in vivo electrophysiology tests or behavioral tests.

### In vivo multi-unit recording

In vivo multiunit recording was performed as previously described [16]. Briefly, animals (both sexes, 10–15 week-old) were implanted with a microarray carrying six tetrodes into the somatosensory cortex, and were subjected to a linear track to record the unit activity. To analyze the level of synchronization of isolated pyramidal neurons, pairs of cells recorded in the same tetrode were subjected to cross-correlation analysis. More details are provided in Supplementary Methods.

### In vivo LFP recording

In vivo LFP recoding was also performed as previously described [17]. Briefly, LFP recording was performed from primary auditory cortex of awake, head-restrained mice (both sexes, 10–15 week-old) in an auditory isolation chamber (background sound level, 35 dB SPL). Five hundred-ms long click trains consisting of 80-dB white-noise pulses presented at 40 Hz (40-Hz click-train stimuli) were applied 50 times with an inter-stimulus interval (ISI) of 20 s. A long ISI was used to robustly evoke the LFP N1 potentials by each click sound (50 times). To measure “evoked ASSR power”, the LFP responses, for each after subtraction of spontaneous LFP at the mid-time point of the preceding ISI, were averaged across 50 click trains, and then spectral analysis on the averaged LFP was performed. To measure “total ASSR power,” spectral analysis was performed on each of LFP traces after subtraction of spontaneous LFP, and then the results of spectra were averaged. Baseline (spontaneous) LFP powers during the pre-stimulus period were also measured during the last 10 s (50 segments of 200-ms bin and averaged) prior to the first click-train stimulus. To calculate the intertrial coherence, phase locking to click-trains was performed in a frequency range 0–100 Hz with 60% overlapping window. Analyses were performed under per-animal design and per-electrode channel design, and male and female data were mixed since no clear sex differences were observed. More details are provided in Supplementary Methods.

### Ex vivo multiple patch clamp recording

To measure spontaneous inhibitory postsynaptic currents (sIPSCs) from pairs of neighboring (<50 µm apart) neurons ex vivo, multiple patch-clamp recordings were performed in auditory cortex layer 2/3 in the presence of CNQX (20 µM) and AP5 (50 µM). Synchronous events were detected by custom written Microsoft Excel (Statcel 4th ed.) macro by defining the sIPSC events (> 5pA) coinciding within ±10 ms of signal window. More details are provided in Supplementary Methods.

### Mouse behavioral tests

Y-maze spontaneous alternation was conducted to assess spatial working memory, as previously described [16]. PPI of acoustic startle was also performed to measure a pre-attentive aspect of cognitive function [30]. Male and female data were mixed because no clear sex difference was observed.

### Statistical analyses

Statistical analyses were conducted using JASP (version 0.12.1) (Univ Amsterdam open-source data analysis software). Student’s *t* test, paired *t* test and factorial analysis of variance (ANOVA) were employed wherever appropriate. When main effects or interaction effects were significant, Tukey-Kramer post hoc analysis was conducted to determine which groups differ significantly from other groups. Data are presented as mean ± s.e.m. Significance was considered at *p* < 0.05.

## Results

### Over-activation of GSK3 in cortical *Grin1*-deleted PV-positive neurons

**Fig. 1:**
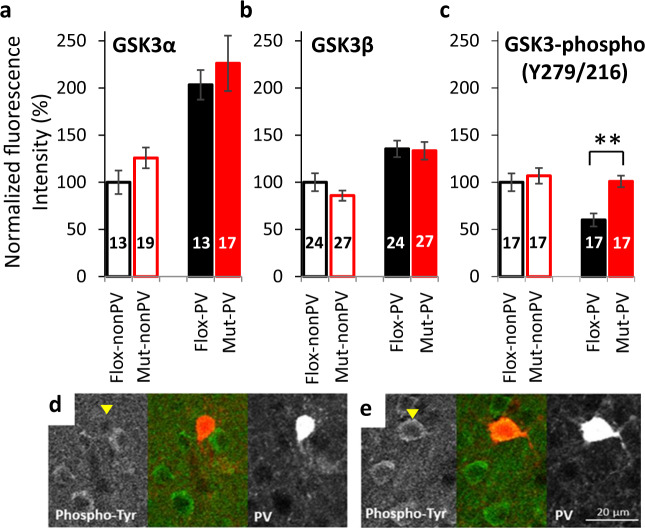
Up-regulation of glycogen synthase kinase 3 (GSK3) in cortical *Grin1*-deleted PV-positive neurons. Immunoreactivity levels in the somata of cortical PV neuron against GSK3α, GSK3β, or phopshorylated-Y216-GSK3β (Y279-GSK3α). Normalized by the average levels of each immunoreactivity (IR) of the non-PV (pyramidal) neurons, and are shown by the level of floxed-*Grin1* control non-PV neurons as 100%. Although there was no difference in GSK3α (**a**) and GSK3β expression levels (**b**), the levels of phosphorylated GSK3 at Tyrosine residues (**c**) were increased in the PV neurons of *Grin1* mutant mice (*p* = 1.01E–04, *t* test), suggesting over-activation of GSK3 in the NMDAR-deleted PV neurons. The number of brain sections obtained from three mice (8–10 week-old, both sexes) per genotype/immunostaining is shown in bar graph. Panels D and E show the images of GSK3 phospho-Tyr immunoreactivity (IR) (Left) and PV-IR (right) of floxed-*Grin1* control mice (**d**) and mutant mice (**e**). Note that GSK3β phospho-Y216 antibody cross-reacts with phospho-Y279 in GSK3α. Arrowheads show cells for double-positive for phosho-Tyr-GSK3 and PV. **p < 0.01.

To explore whether GSK3 is up-regulated in the *Grin1* mutant mice, we measured fluorescence intensity of auditory cortical neurons produced by fluorescent-labeled antibodies against GSK3α, GSK3β, and phospho-GSK3 (at Y279 for GSK3α and at Y216 for GSK3β), an auto-activated form of GSK3 [31, 32], respectively (Fig. 1). Although the expression of GSK3α (Fig. 1a) and GSK3β (Fig. 1b), both, are higher in the cortical layer 2/3 PV neurons compared to the nearby non-PV neurons (largely pyramidal neurons), no difference in the protein levels was detected between the genotypes in either PV neurons or non-PV neurons. However, tyrosine phosphorylation at Y279/Y216 in GSK3α/β was significantly intensified in the PV neurons of mutant mice (increased by 40%), compared to the PV neurons of floxed control mice (Fig. 1c), suggesting that NMDAR deletion in PV neurons leads to over-activation of GSK3 in the PV neurons.

### Non-selective GSK3 inhibitor reversed stimulus-evoked gamma oscillation deficits, spontaneous gamma oscillation and cognitive dysfunction in *Grin1* mutant mice

**Fig. 2:**
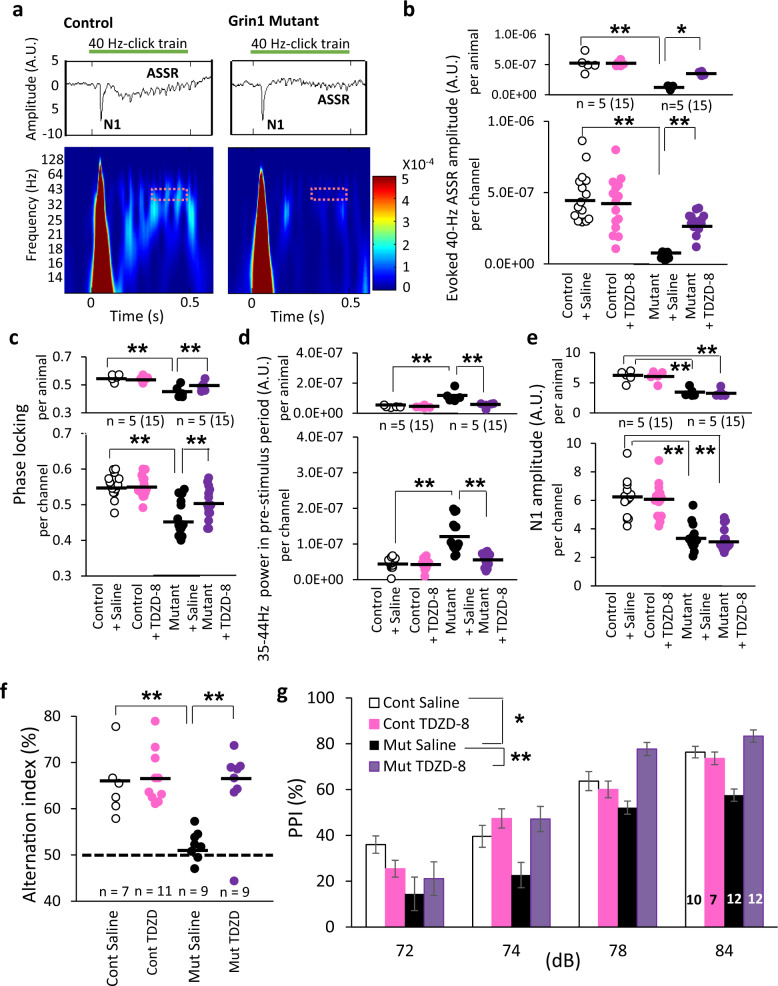
Non-selective GSK3 inhibitor alleviated in vivo stimulus-evoked gamma oscillation deficits and cognitive dysfunction in *Grin1* mutant mice. **a** Examples of the averaged ASSR LFP trace (middle, *z*-score) and its evoked power spectrogram (bottom) in response to 40-Hz click trains (upper; 80 dB intensity, 500 ms duration) from a floxed-control mouse (left) and a *Grin1* mutant mouse (right). Time 0 is tone onset. The red dashed area (35–44 Hz frequency and during 200 ms before cessation of the click train) was for the following ASSR analyses. **b** Evoked–power amplitudes at 35–44 Hz during 200 ms before cessation of click-train stimuli were obtained by time-frequency decomposition of averaged LFP trace across 50 trains of stimuli following subtraction of spontaneous power amplitude (*z*-score, 200-ms segment) at the mid-time point of the ISIs (20-s duration) from LFP power amplitude (z-score). Evoked power amplitudes were evaluated before and after *i.p*. injection of TDZD-8 (non-selective GSK3 inhibitor) in control (15 channels) and mutant mice (15 channels) per animal (top) and per channel (bottom) [before TDZD-8 treatment, controls vs mutants, F(1,28) = 217.2, drug × genotype, *p* < 0.001 (per channel), F(1,8) = 3.33, *p* < 0.001 (*p*er animal), two-way Repeated Measures ANOVA with Tukey-Kramer post hoc test]. TDZD-8 normalized ASSR power at 40 Hz in *Grin1* mutant mice [*p* < 0.001 (per channel), *p* = 0.049 (per animal), two-way Repeated Measures ANOVA with Tukey-Kramer post hoc test], but not in the control mice [*p* = 1.00 (per channel), *p* = 0.95 (per animal)]. No difference between TDZD-8 treated mutant and control mice per channel (*p* = 0.09, fully rescued). **c** Phase locking to 40-Hz click-train stimuli before and after TDZD-8 treatment also partly reversed the phase locking deficits to 40 Hz click-train stimuli in *Grin1* mutant mice per channel (bottom) [before vs after treatment, F(1,28) = 18.8, drug × genotype, *p* < 0.001 (per channel), F(1,8) = 30.3, *p* < 0.001 (per animal), two-way repeated measures ANOVA with Tukey-Kramer post hoc test], but not in control mice [*p* = 0.97 (per channel), *p* = 0.63 (per animal)]. No difference between TDZD-8 treated mutant and control mice per animal (*p* = 0.11, fully rescued). **d** Elevated baseline power at 35–44 Hz in the pre-stimulus period was normalized by TDZD-8 in *Grin1* mutant mice saline vs TDZD-8, drug × genotype, F(1,28) = 33.7, *p* < 0.001 (per channel), F(1,8) = 13.1, *p* < 0.01 (per animal), two-way Repeated Measures ANOVA with Tukey-Kramer post hoc test. No difference between TDZD-8 treated mutant and control mice per channel (*p* = 0.56) and per animal (*p* = 0.75, fully rescued). **e** TDZD-8 did not affect the N1 amplitudes elicited by the click-train stimuli in *Grin1* mutant mice although the N1 amplitudes were lower in the mutant mice compared to floxed-controls (controls vs mutants, *p* < 0.001 (per channel), *p* < 0.01 (per animal), two-way Repeated Measures ANOVA with Tukey-Kramer post hoc test). **f** In Y-maze spontaneous alternation task, the alternation index of the mutant mice was near the chance level (50% dotted line), suggesting spatial working memory deficit (control mice vs original *Grin1* mutants, F(1,32) *=* *8.35*, *p* < 0.005, one-way ANOVA with Tukey-Kramer post hoc test). The same index in a different cohort of mutants was returned back to the level (over 65%) of control mice one hr after TDZD-8 administration (mutants with TDZD-8 vs mutants with saline, *p* < 0.005, one-way ANOVA with Tukey-Kramer post hoc test). TDZD-8 did not affect the alternation index of the control mice (controls with TDZD-8 vs controls with saline, *p* = 1.00, one-way ANOVA with Tukey-Kramer post hoc test). **g** TDZD-8 reversed prepulse inhibition (PPI) deficits of startle reflex across prepulse intensities (TDZD-8 vs saline, F(3,111) = 3.94, *p* < 0.005, Repeated Measures ANOVA, Tukey-Kramer post hoc test). TDZD-8 showed no impact on PPI of the control mice (TDZD-8 vs saline, *p* = 0.97, Repeated Measures ANOVA, Tukey-Kramer post hoc test). **p < 0.01 and *p < 0.05, black line shows average. Each dot indicates the individual data per animal or per channel. The number of channel is shown in parentheses next to the number of animal.

To assess the impact of predictive GSK3 over-activity on the in vivo stimulus-evoked LFP gamma oscillations, we used a non-selective GSK3 inhibitor, TDZD-8. Before TDZD-8 administration, ASSRs were measured from the *Grin1* mutant mice and floxed-control mice during 40-Hz click trains (Fig. 2a). As previously reported [17], 40-Hz click trains evoked the N1 potential (defined as the amplitude of the first prominent negative peak) followed by the robust increase in the power magnitudes, in particular at low gamma frequency range, in the floxed-*Grin1* control mice (Fig. 2a). Genotypic comparison revealed a profound reduction of the evoked ASSR power amplitudes at 40-Hz in the *Grin1* mutant mice (Fig. 2a, b). One hr after administration of TDZD-8, the evoked powers were restored in the mutant mice whereas no drug effect was observed in controls (Fig. 2b). The analysis of total ASSR power using the same LFPs reach the same conclusions (Supplementary Fig. S3A and Supplementary Result and Discussion). In addition, we found that the degree of phase locking in the ASSR to 40-Hz click trains was lower in the mutants, which was also reversed in the mutant mice in per-channel analysis, while being unaffected in the floxed-control mice (Fig. 2c). TDZD-8 also normalized abnormally high baseline gamma power amplitudes during the last 10 s prior to the onset of the first click-train (referred to a pre-stimulus period), to the baseline level of control mice (Fig. 2d). On the other hand, the N1 amplitudes evoked by click trains were smaller in the mutant mice, which were unaffected by TDZD-8 (Fig. 2e). These results suggest that acute GSK3 inhibition alleviates the evoked-power deficits and reversed the baseline gamma power in the pre-stimulus period of the mutant mice.

We also assessed whether TDZD-8 restored the normal behavior in the *Grin1* mutant mice. TDZD-8 restored the Y-maze spontaneous alternation, which was diminished in the mutant mice as previously described [16] (Fig. 2f). Pretreatment with TDZD-8 also alleviated the deficits in PPI of startle in the mutant mice (Fig. 2g). These results suggest that acute GSK3 inhibition ameliorates cognitive dysfunction of the *Grin1* mutant mice.

### Paralog-selective GSK3β inhibitor, but not GSK3α inhibitor, normalized the deficits in gamma oscillation and cognitive dysfunction of *Grin1* mutant mice

**Fig. 3:**
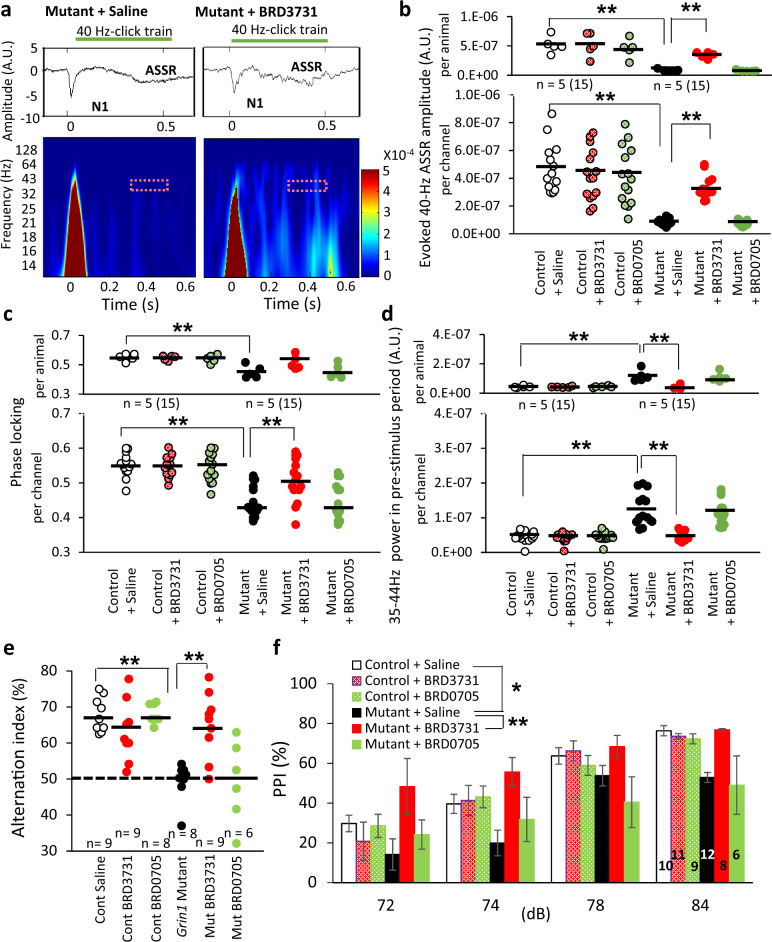
Paralog-selective GSK3β inhibitor (BRD3731), but not GSK3α inhibitor (BRD0705), alleviated gamma oscillation deficits and cognitive dysfunction in *Grin1* mutant mice. **a** Representative examples of the averaged 40 Hz ASSR (middle, z-score) and spectrogram (bottom) in response to 40 Hz click trains (upper; 80 dB intensity, 500 ms duration) from a mutant mouse with saline injection (left) and a mutant mouse with BRD3731 injection (right). Time 0 is tone onset. The red dashed area (35–44 Hz frequency and during 200 ms before cessation of the click train) was for the following ASSR analyses. **b** Stimulus-evoked 40-Hz ASSR before and after *i.p*. injection of either BRD3731 or BRD0705 in floxed-control (15 channels) and mutant mice (15 channels). BRD3731, but not BRD0705, alleviated ASSR power deficits at 40 Hz in *Grin1* mutant mice by per-animal and per-channel analyses [15 channels before and after BRD3731, F(2,84) = 7.12, drug × genotype*, p* < 0.01 (per channel), F(2,24) = 3.01*, p* < 0.01 (per animal); 15 channels before and after BRD0705, *p* = 0.998 (per channel), *p* = 1.00 (per animal), two-way ANOVA with Tukey-Kramer post hoc test]. No difference between BRD3731-treated mutant and control mice per channel (*p* = 0.75) and per animal (*p* = 0.23, fully rescued). Neither BRD3731 nor BRD0705 affected the evoked-gamma oscillation in floxed-control mice (before and after BRD3731, *p* = 0.93; before and after BRD0705, *p* = 0.57, two-way ANOVA with Tukey-Kramer post hoc test). **c** BRD3731, but not BRD0705, also alleviated the impaired phase locking to 40 Hz click-train stimuli in *Grin1* mutant mice (15 channels before and after BRD3731, F(2,84) = 4.88, drug × genotype*, p* < 0.01 (per channel), F(2,24) = 2.40*, p* = 0.10 (per animal); 15 electrode channels before and after BRD0705, *p* = 1.00 (per channel), *p* = 1.00 (per animal), two-way ANOVA with Tukey-Kramer post hoc test). No difference between BRD3731-treated mutant and control mice per channel (*p* = 0.12) and per channel (*p* = 0.74, fully rescued). **d** BRD3731, but not BRD0705, normalized the baseline gamma power in the pre-stimulus period in *Grin1* mutant mice by both per-animal and per-channel analyses (BRD3731 vs saline, 15 electrode channels, F (2,84) = 17.7, drug × genotype*, p* < 0.001 (per channel), F(2,24) = 5.74*, p* < 0.001 (per animal), BRD0705 vs saline, 15 electrode channels, *p* = 0.93 (per channel), *p* = 0.99 (per animal), two-way ANOVA with Tukey-Kramer post hoc test). No difference between BRD3731-treated mutant and control mice per channel (*p* = 1.00) and per animal (*p* = 1.00, fully rescued). **e** In Y-maze spontaneous alternation task, the alternation index of the mutant mice was near the chance level, suggesting spatial working memory deficit (floxed-control mice vs *Grin1* mutants, F(5,42) = 9.96, *p* < 0.001, one-way ANOVA with Tukey-Kramer post hoc test). The same index in a different cohort of mutants was returned back to over 65% level of control mice one hr after *i.p*. administration of BRD3731 (mutants with BRD3731 vs mutants with saline, *p* < 0.005, one-way ANOVA with Tukey-Kramer post hoc test), but not BRD0705 (mutants with BRD0705 vs mutants with saline, *p* = 1.00, one-way ANOVA with Tukey-Kramer post hoc test). Neither BRD3731 nor BRD0705 affected the alternation index of control mice (controls with BRD3731 vs controls with saline, *p* = 0.77, controls with BRD0705 vs controls with saline, *p* = 1.00, ANOVA with Tukey-Kramer post hoc test). **f** BRD3731 reversed PPI deficits of startle across the prepulse intensities in *Grin1* mutant mice. (BRD3731 vs saline, F(3,138) = 1.69, *p* < 0.05, Repeated Measures ANOVA Tukey-Kramer *post hoc* test), but not BRD0705 (*p* = 1.00). Neither BRD3731 nor BRD0705 had impact on PPI of control mice (BRD3731 vs saline, *p* = 1.00, BRD0705 vs saline, *p* = 1.00). **p < 0.01 and *p < 0.05, black line shows average. Each dot indicates the individual data per animal or per channel. The number of channel is shown in parentheses next to the number of animal.

To determine the over-activation of which isoform, GSK3α or GSK3β, elicits the gamma oscillation deficits, we used a paralog-selective GSK3β inhibitor BRD3731 and a GSK3α inhibitor BRD0705. Administration of BRD3731, but not BRD0705, reversed the gamma oscillation deficits in the mutant mice (Fig. 3a, b). On the other hand, neither BRD3731 nor BRD0705 affected the evoked-gamma oscillation in the floxed-control mice. The total ASSR power analysis reach the same conclusion (Supplementary Fig. S3B). In addition, administration of BRD3731, but not BRD0705, also augmented phase locking factor at 40 Hz in the mutant mice (Fig. 3c). Pretreatment with BRD3731, but not BRD0705, suppressed abnormally high mutants’ baseline gamma and beta power to the normal level in the pre-stimulus period (Fig. 3d and Supplementary Fig. S5). On the other hand, neither BRD3731 nor BRD0705 affected the N1 amplitudes of the mutant mice, nor any parameters in the floxed-control mice (Supplementary Fig. S4A). Finally, BRD3731, but not BRD0705, rescued the spatial working memory of *Grin1* mutant mice in Y-maze alternation task (Fig. 3e). Pretreatment with BRD3731, but not BRD0705, also ameliorated the mutants’ PPI deficits (Fig. 3f). The startle reflex itself was unaffected by either genotypes or drug treatments (Supplementary Fig. S5A). These results suggest that GSK3β inhibition, but not GSK3α inhibition, is crucial for restoration of gamma oscillations and cognitive function.

### GABAergic neuron-selective *GSK3B* knockdown reverses stimulus-evoked gamma oscillation deficits, excessive baseline gamma oscillations and cognitive dysfunction

**Fig. 4:**
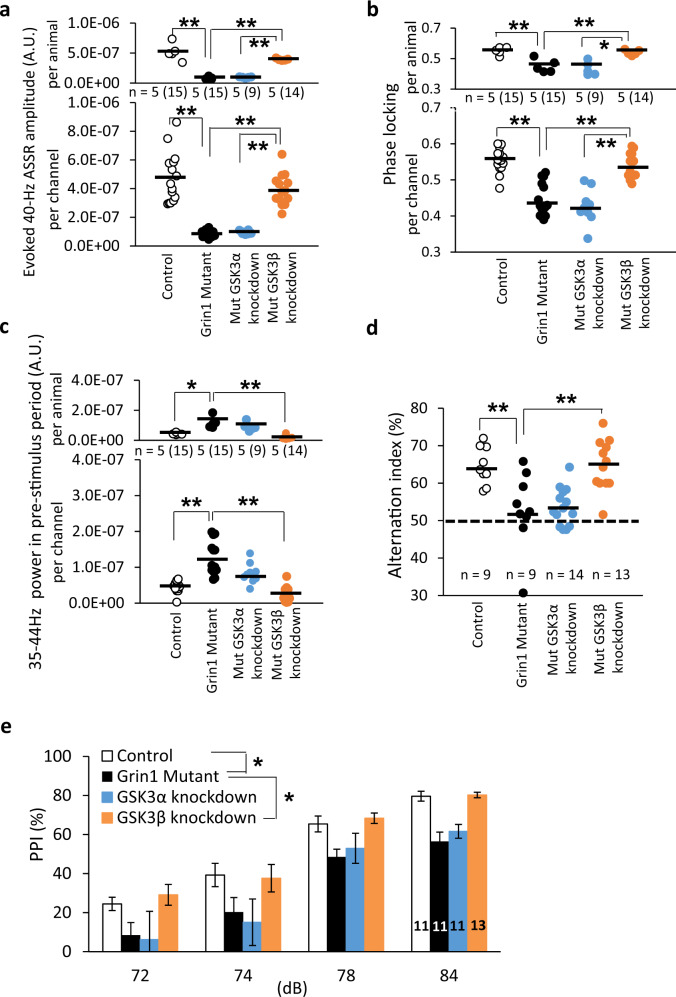
GABAergic neuron-selective *GSK3B* knockdown restored diminished stimulus-evoked gamma oscillations and cognitive behaviors. **a** Stimulus-evoked 40-Hz ASSR amplitudes (as determined by subtraction of spontaneous power amplitude (200-ms segment) in the 20-s long ISI from total ASSR power amplitude during 200 ms before cessation of the click-train) were evaluated in floxed-control mice (15 channels), *Grin1* mutant mice (15 channels), *GSK3A* knockdown mice on *Grin1* mutant background and *GSK3B* knockdown mice on the mutant background (14 channels). *GSK3B* knockdown in *Grin1* mutant mice, but not *GSK3A* knockdown, normalized 40-Hz ASSR power assessed both by per-animal (top) and per-channel (bottom) designs (GSK3B knockdown vs original *Grin1* mutant mice, F(3,49) = 7.54, *p* < 0.001 (per channel), F(3,16) = 45.8, *p* < 0.001 (per animal), one-way ANOVA with Tukey-Kramer post hoc test). No difference between *GSK3B* knockdown and control per channel (*p* = 0.18) and per animal (*p* = 0.061, fully rescued). **b** The degree of Phase locking to 40-Hz click-train stimuli was analyzed in control, *GSK3A* knockdown and *GSK3B* knockdown in the Grin1 mutants. *GSK3B* knockdown, but not *GSK3A* knockdown, rescued the impaired phase locking to 40-Hz stimuli (GSK3B knockdown vs original *Grin1* mutant mice, F(3,49) = 33.4, *p* < 0.001 (per channel), F(3,16) = 15.1, *p* < 0.01 (per animal), one-way ANOVA with Tukey-Kramer post hoc test). No difference between *GSK3B* knockdown and control per channel (*p* = 0.76) and per animal (*p* = 0.95, fully rescued). **c** *GSK3B* knockdown, but not *GSK3A* knockdown, reversed the elevated baseline power at 35–44 Hz in the pre-stimulus period (F(3,49) = 27.3, *p* < 0.001 (per channel), F(3,16) = 14.4, *p* < 0.001 (per animal), one-way ANOVA with Tukey-Kramer post hoc test). No difference between *GSK3B* knockdown and control per channel (*p* = 0.24) and per animal (*p* = 0.49, fully rescued). **d** In Y-maze spontaneous alternation task, the alternation index of the *Grin1* mutant mice was near the 50% chance level control mice vs original *Grin1* mutants (F(3,41) = 10.6, *p* < 0.01, one-way ANOVA with Tukey-Kramer *post hoc* test). *GSK3B* knockdown, but not *GSK3A* knockdown, augmented the index close to control mice (*p* < 0.001, one-way ANOVA with Tukey-Kramer post hoc test). **e** *GSK3B* knockdown, but not *GSK3A* knockdown, normalized the PPI of startle across prepulse intensities compared to the *Grin1* mutant mice (*GSK3B* knockdown vs for original mutants, F(3,126) = 104.1, *p* < 0.05, Repeated Measures ANOVA Tukey-Kramer *post hoc* test). **p < 0.01 and *p < 0.05, black line shows average. Each dot indicates the individual data per animal or per channel. The number of channel is shown in parentheses next to the number of animal.

To address whether GABAergic neuron-specific GSK3β inhibition is sufficient for restoration of gamma oscillations and cognitive behavior, we bred either a floxed-GSK3A or a floxed-GSK3B mouse strain to the Ppp1r2cre/*Grin1* KO mice to produce the corticolimbic GABAergic neuron-selective heterozygous deletion of *GSK3A* or *GSK3B* gene, respectively, on *Grin1* mutant genetic background (GABAergic neuron-selective knockdown). Genetic *GSK3B* knockdown, but *GSK3A* knockdown, fully alleviated the defective gamma power amplitudes (Fig. 4a and Supplementary Fig. S3C) and phase locking factors at 40 Hz of the *Grin1* mutant mice (Fig. 4b). *GSK3B* knockdown, but not *GSK3A* knockdown, also fully normalized baseline gamma oscillation during the pre-stimulus period (Fig. 4c). However, neither genetic *GSK3A* nor *GSK3B* knockdown altered the N1 amplitudes (Supplementary Fig. S3D). We also examined whether knockdown of GSK3-isoform restores the cognitive behavior. Genetic *GSK3B* knockdown, but not *GSK3A* knockdown, improved the Y-maze spontaneous alternation (Fig. 4e) and PPI of the *Grin1* mutant mice (Fig. 4f). No difference was detected in the startle amplitudes between the genotypes (Supplementary Fig. S5B). These results suggest that GSK3β inhibition in corticolimbic GABAergic neurons is sufficient for restoration of gamma oscillations and cognitive function.

### GABAergic neuron-selective *GSK3B* knockdown alleviated in vivo spike synchrony and ex vivo synchronous sIPSC deficits

**Fig. 5:**
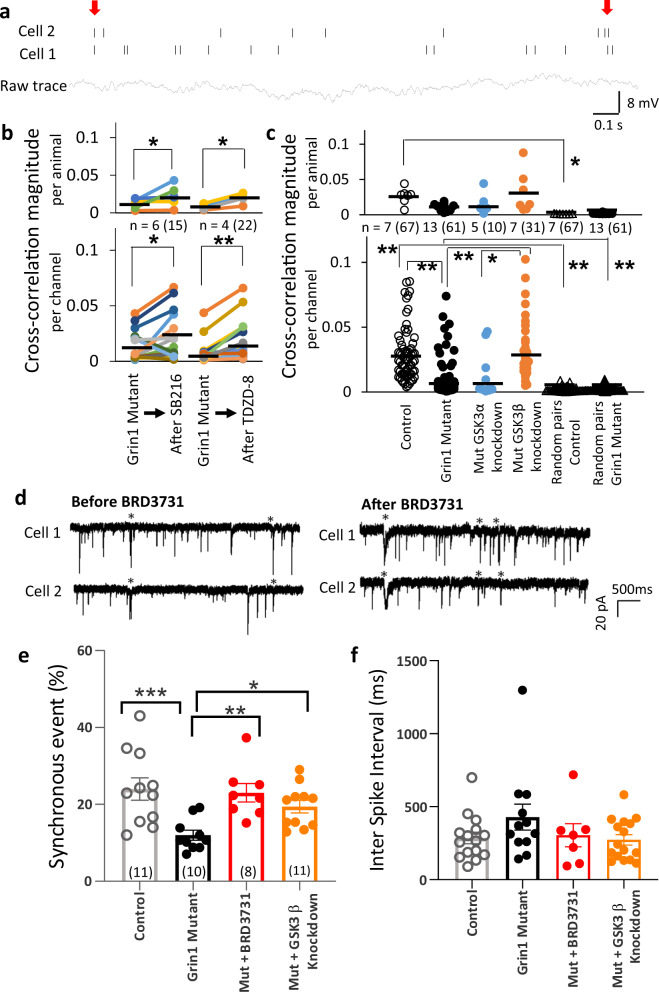
Selective *GSK3B* knockdown in GABAergic neurons increases in vivo action potential synchrony and ex vivo synchronous sIPSCs from pairs of cortical pyramidal cells in *Grin1* mutant mice. **a** Representative raw trace of multi-unit recording from somatosensory cortex from floxed-control mouse (bottom). After spike sorting, two pyramidal cell activities are shown (top), which was used for cross-correlation analysis. Red arrows indicate the synchronous unit activities of the paired pyramidal cells. **b** Plots of cross-correlation magnitudes before and after treatment with GSK3 inhibitor. The deficits of in vivo spike synchrony in *Grin1* mutants disappeared 1 h after *i.p*. treatment with GSK3 inhibitor TDZD-8 and SB216763 (SB216) by both per-animal (top) and per-channel (bottom) analyses (SB216763: 15 pairs, *p* < 0.05, paired *t* test, TDZD-8; 21 pairs *p* < 0.0005, paired *t* test). **c** The degree of cross-correlation of in vivo spike firing of pairs of pyramidal cells in somatosensory cortex was much lower in *Grin1* mutant mice (61 pairs) compared to floxed-control mice (67 pairs). In vivo spike synchrony was restored when *GSK3B* was heterozygously knocked-out (knockdown) in *Grin1*-deleted GABAergic neurons (31 neuron-pairs from *GSK3B* knockdown mice vs 61 pairs from the original *Grin1* mutants, F(5,290) = 43.4, *p* < 0.001, one-way ANOVA with Tukey-Kramer post hoc test); but not when *GSK3A* was knockdown (10 pairs from *GSK3A* knockdown mice vs 61 pairs from original *Grin1* mutants, *p* = 0.99, one-way ANOVA with Tukey-Kramer post hoc test). Cross-correlation coefficient of mutants was higher than when the same spike trains data set was randomly shuffled, suggesting that low cross-correlation coefficient of mutants is not due to a random temporal overlap of spike trains from their pyramidal cell pairs. Each dot indicates the individual data per animal or per channel. The number of channel is shown in parentheses next to the number of animal. **d** Representative traces showing spontaneous IPSC (sIPSC) activity ex vivo, simultaneously recorded from a pair of layer 2/3 pyramidal cells from auditory cortex of the *Grin1* mutant mice before and after BRD3731 application, synchronized events are indicated by asterisks. **e** Ex vivo whole-cell patch-clamp recording from pairs of neighboring pyramidal cells in cortical layer 2/3 showed reduced synchronous sIPSC events in *Grin1* mutants compared to controls [23.97 ± 2.9% for floxed-controls (*n* = 11 pairs) vs 11.97 ± 1.3% for mutants (*n* = 10 pairs), ***p < 0.001]. This decrease was reversed by application of BRD3731 (23.01 ± 2.4%, *n* = 8 pairs) to *Grin1* mutants and in *GSK3B* knockdown on the mutant background (19.43 ± 1.65%, *n* = 11 pairs). **p < 0.01 and *p < 0.05. **f** Summary plots of inter-event intervals of sIPSCs under the given experimental conditions (286.3 ± 39.6 ms for floxed-control; vs 429.0 ± 89.0 ms for *Grin1* mutant, *p* = 0.19; mutant vs mutant with BRD3731 application: 304.8 ± 79.2 ms, *p* = 1.00; mutant vs GSK3B knockdown on mutant background, 273.4 ± 35.1 ms, *p* = 1.00). Student’s *t* test.

To assess whether GSK3 inhibitors impact on spike synchrony in vivo, we measured multi-unit activity from somatosensory cortex in the *Grin1* mutant mice before and after drug treatment. Individual spikes were obtained from nearby pyramidal neurons through the tetrodes’ cell-cluster analysis during simple linear track exploration (Fig. 5a). As previously reported [16], the *Grin1* mutant mice showed much lower correlation coefficients of spikes from pairs of pyramidal neurons compared to the control mice (Fig. 5c). Pretreatment with the non-selective GSK3 inhibitor SB216763 or TDZD-8 both increased in vivo spike synchrony as measured by cross-correlation magnitude, in the *Grin1* mutant mice (Fig. 5b). To determine which isoform of GSK3 results in vivo spike synchrony defects, multi-unit recording from somatosensory cortex was also performed in the GABAergic neuron-selective GSK3α and GSK3β knockdown mice. As shown in Fig. 5c, in vivo spike synchrony was restored in genetic GSK3β knockdown animals, but not in GSK3α knockdown animals.

In another series of experiments, we measured spontaneous IPSCs (sIPSCs) from pairs of layer 2/3 pyramidal neurons of auditory cortex. Ex vivo recordings were obtained in brain slices prepared from floxed-control and *Grin1* mutant mice (Fig. 5d). The *Grin1* mutant mice showed a significant reduction in synchronous sIPSC events between pairs of simultaneously recorded pyramidal neurons (Fig. 5e), suggesting an impairment in synchronous GABA release from *Grin1*-deleted neurons. Notably, synchronous events of sIPSCs were significantly increased to the levels of controls by bath-application of BRD3731 (40 µM). In addition, in GABAergic neuron-selective *GSK3B* knockdown animals, the number of synchronous events in layer 2/3 pyramidal cells was equivalent to the number of control mice. Inter-event intervals of sIPSCs were not significantly altered across groups (Fig. 5f), suggesting that synchronous GABA release is disturbed by *Grin1*-deletion in the GABAergic neurons.

## Discussion

We demonstrated that GSK3β inhibition restores the in vivo cortical gamma oscillation and cognitive function in the mouse model of *Grin1* hypofunction relevant to schizophrenia. Specifically, we found that (1) elevated phospho-GSK3 (at Y216 in GSK3β) immunoreactivity in the PV neurons (presumably *Grin1*-deleted), but not pyramidal neurons, in the auditory cortex of the *Grin1* mutant mice, (2) normalization of the diminished evoked-gamma oscillations, abnormally high baseline gamma oscillation, and behavioral deficits in the *Grin1* mutant mice by administration of non-selective GSK3 inhibitors, (3) reversal of the evoked-gamma oscillations, baseline gamma power, spatial working memory and PPI by paralog-selective GSK3β inhibitor (BRD3731), but not by GSK3α inhibitor (BRD0705), and (4) restoration of evoked-gamma oscillations, baseline gamma power, and cognitive behaviors by corticolimbic GABAergic neuron-selective *GSK3B* genetic knockdown. These findings suggest that GSK3β inhibition in corticolimbic GABAergic neurons rescues cognitive behavior in the NMDAR hypofunction mouse model, in strong association to the restoration of normal synchronous oscillatory brain activity at gamma frequency both in the stimulus and pre-stimulus period. Consistently, in vivo deficits in spike synchrony from pairs of cortical layer 2/3 pyramidal neuron was reversed by GSK3β inhibition and by GABAergic neuron-specific knockdown of *GSK3B*, but not *GSK3A*. Ex vivo reduction of synchronous sIPSC event number in pairs of pyramidal neurons were also restored by GSK3β inhibition and by GABAergic neuron-selective *GSK3B* genetic knockdown, suggesting that evoked-gamma oscillation deficit in the *Grin1* mutant mice is attributed to the synchronous GABA release impairment and subsequent pyramidal cells’ spike synchrony deficits.

### Two distinct gamma power abnormalities originating from *Grin1*-deleted PV neurons

Abnormal cortical gamma oscillatory activity appears to underlie impairment in higher cognitive functions associated with schizophrenia. Originating from the landmark observation by Kwon et al. [3], several groups have confirmed the reduction of the evoked power and synchronization to 40-Hz click-train stimuli in patients with schizophrenia [33–35]. On the other hand, spontaneous gamma power has also been reported to increase during the pre-stimulus baseline period in patients experiencing positive auditory symptoms [6] or during the resting state [7, 8]. However, it has been debated whether the mechanisms of producing such two gamma abnormalities are shared or distinct. We previously demonstrated that *Grin1* deletion in cortical GABAergic neurons elicits such dual gamma abnormalities in the same animal [17]. We postulated that the evoked gamma power deficits could be due to the impaired feed-forward inhibition of thalamo-cortical circuits in response to acoustic stimuli by the defective cortical PV neurons, which may attenuate the phase-locked firing of pyramidal neurons at gamma frequency range [17]. On the other hand, the reason for the elevated baseline gamma power may be attributable to the aberrant or “noisy” spike firing of pyramidal neurons, due to the cortical disinhibition by defective PV neurons. The present study corroborates this prediction and further suggests a shared underlying mechanism between the two, because both abnormalities are consistently reversed together upon various GSK3β activity manipulations. The responsible cell-type is likely to be PV neurons, because PV neuron-specific *Grin1* deletion in mice results in optogenetically-evoked gamma oscillation deficits and increased baseline power [18, 19]. In the *Grin1* mutant mice of this study, a majority (75–84%) of cortical PV neurons is *Grin1*-deleted, whereas Cre recombination frequency is much lower in other PV-negative GABAergic neurons [16].

Curiously, the spontaneous gamma power (200-ms segment) at the mid-time point of ISIs was normal (in z-score) and unaffected by the GSK3β inhibition. Since auditory layer 2/3 PV neurons are consistently suppressed in active wakefulness by acoustic stimuli [36], functional insufficiency of *Grin1*-deleted PV neurons may be masked when click-trains are being applied [17]. Alternatively, long ISI (20 s) used in this study might decay the noise level at the mid-time point of ISI. An increase in induced gamma power has been reported in schizophrenia in relatively short ISIs (~1–3 s) during auditory steady-state stimulation [37–39]. Regardless, it suggests that the evoked-gamma power deficit is not simply caused by elevation of spontaneous power during the click-train stimulus period.

### From *Grin1* deletion in PV neuron to evoked-gamma power and phase deficits

Axons of a single PV neuron in the cortex and hippocampus innervate hundreds of pyramidal cells [40]. It has been reported that sIPSCs between pairs of nearby pyramidal cells in mouse entorhinal cortex are highly correlated [41]. Indeed, the simultaneous occurrence of multiple sIPSCs among cortical layer2/3 pyramidal neurons was frequently detected in the floxed-control mice (Fig. 5d). However, such synchronous sIPSCs were less frequent in the *Grin1* mutant mice, which was reversed by the selective GSK3β inhibitor and GABAergic neuron-selective GSK3 knockdown (Fig. 5e). These findings are consistent with a prevailing hypothesis that gamma oscillation abnormalities are attributable to impaired GABAergic neurotransmission [42, 43]. Further investigation is warranted, however, to determine the underlying mechanism(s) of synchronous GABA release, as GABA release frequency appeared to be unaffected (Fig. 5f).

Anyhow, such impairment of synchronous perisomatic inhibition onto pyramidal neurons could lead to in vivo spike synchrony deficit in *Grin1* mutant mice. This is presumably because PV-positive basket cells predominantly innervate to the soma and proximal dendritic areas of pyramidal cells [44], providing a robust perisomatic inhibition, thereby controlling spike generation [45]. Subsequently, spike synchrony deficits could give rise to impairments in synchronized LFP oscillations to the 40-Hz click-train stimuli, because LFP oscillations are at least partly attributed to the synchronization of neuronal firing rate on the same time scale [46].

### Disturbance of gamma oscillation leading to cognitive deficits

One potential approach to assess the relation between gamma oscillation abnormalities and cognitive dysfunction is to explore whether reversing the gamma abnormalities could lead to the restoration of cognitive function. Enhanced GABAergic activity with the potentiator of α2 subunit containing GABA_A_ receptors improved frontal gamma oscillatory activity and working memory in patients with schizophrenia [47]. Preclinically, GABA_B_ receptor agonist baclofen has suggested to improve gamma synchrony and spatial working memory deficits in the NMDAR hypomorph mice [48]. Recently, GSK3 inhibition has been shown to rescue the deficits in spatial working memory and in phase-locking of mPFC activity to theta/gamma oscillations in the ventral hippocampus of 22q11.2 microdeletion model mice [24].

### Limitations

We repeatedly showed that systemic or GABAergic neuron-selective GSK3β inhibition restores the in vivo spike synchrony, gamma oscillations and cognitive behaviors altogether, suggesting that spike synchrony, gamma oscillation and cognitive function are tightly associated. However, it should be noted that, while LFP gamma oscillations were assessed in the auditory cortex and in vivo spike synchrony in somatosensory cortex, the behavioral tests assessed may be dependent on other brain regions, such as the prefrontal cortex and/or hippocampus [49, 50]. Thus, a direct link has not been established between evoked-gamma oscillations and cognitive function. Consistent demonstration of reversal responses by GSK3 inhibition among the manipulations may suggest that restoration of gamma oscillations and spike synchrony elicited by GSK3β inhibition at PV-positive interneurons may constitute a shared mechanism across the cortical areas.

Secondly, this study involved a relatively small sample size (five mice for each genotype) owing to the difficulty in recording the stimulus-evoked LFPs from mouse primary auditory cortex [17]. However, the results under per-animal analysis design were fully replicated by under per-channel analysis design, suggesting the robustness of the findings.

In summary, we demonstrated a strong association between the integrity of cortical gamma oscillations and cognitive function, by manipulating GSK3β activity. The precise cellular mechanism regarding how GSK3β kinase inhibition ameliorates the synchronous GABA release, in vivo spike synchrony and LFP gamma oscillations has remained unsolved. However, impaired synchronous inhibition of pyramidal neurons by *Grin1*-deleted PV neurons appears to be a key mechanism underlying schizophrenia-like phenotypes. Further study is warranted towards potential development of therapeutic interventions of schizophrenia.

## Funding and disclosure

This work was funded by grants from the National Institute of Mental Health R01 MH110681 (KN) and partly by Change Campaign Fund at Southern Research. The authors report no biomedical financial interests or potential conflicts of interest.

## Supplementary information

Supplementary Information
