# Supplementary material for: GSK3β inhibition restores cortical gamma oscillation and cognitive behavior in a mouse model of NMDA receptor hypofunction relevant to schizophrenia
Source: Neuropsychopharmacology. 2020 Aug 28;45(13):2207–18. doi: 10.1038/s41386-020-00819-0 (PMC7784891; doi:10.1038/s41386-020-00819-0)
Supplement: Supplementary file 1 — Supplementary Information [file 41386_2020_819_MOESM1_ESM.pdf]

GSK3β inhibition restores cortical gamma oscillation and cognitive behavior in a mouse model of NMDA receptor hypofunction relevant to schizophrenia

Kazuhito Nakao[a,b], Mahendra Singh[a], Kiran Sapkota[a], Bailey C Hagler[a], Robert N Hunter[c], Chander Raman[d], John J Hablitz[e] and Kazu Nakazawa[a,]

[a]*Department of Neuroscience, Southern Research, Birmingham, AL, USA 35205*
[b]*Department of Psychiatry and Behavioral Neurobiology, University of Alabama at Birmingham, Birmingham, AL, USA 35294*
[c]*Department of Chemistry, Southern Research, Birmingham, AL, USA 35205*
[d]*Department of Medicine, University of Alabama at Birmingham, Birmingham, AL, USA 35294*
[e]*Department of Neurobiology, University of Alabama at Birmingham, Birmingham, AL, USA 35294*

Supplementary Information

Supplementary Methods

In vivo LFP recording

Animals were anesthetized with isoflurane to surgical levels and were mounted in a stereotaxic instrument with non-rupture ear bars (Zygoma ear cups, David Kopf Instruments). A custom-made plastic headpost was secured to the occipital bone at the midline with superglue and dental acrylic, and was used to fix the animal's skull to the stereotaxic instrument. This was done to prevent physical occlusion of the external ear canals by stereotaxic ear bars in order to obtain auditory-evoked local field potential (LFP) responses. The microwire multi-electrode array consisted of six tetrodes, which were custom-configured in a 2 × 3 matrix with inter-electrode distance of ~200 µm, covering 0.6 × 0.8 mm^2. The impedance of each electrode was between 0.2 and 0.3 MΩ. The microwire array was inserted into the superficial layers of primary auditory (A1) cortex (2 mm lateral and 1 mm posterior from bregma) at an angle of 5°, and two stainless steel screws in the frontal cortex which served as ground and reference electrodes. After the dosage of isoflurane was reduced to 1%, a single white-noise pulse (1 ms, duration; 80 dB, SPL) was applied to activate A1 cortical area. Since high dose of isoflurane is known to suppress the evoking responses, it is critical to maintain the isoflurane concentration at 1% [1]. The animal was held in place with adhesive tape to prevent head twitching or grooming. If click train-evoked potentials were detected in at least one electrode of the microwire array, the electrodes were moved down slightly to obtain maximal responses in more than one electrodes. The anesthetic dose was then returned back to surgical levels, and the microwire array was fixed to the skull with dental acrylic.

Seven days after surgery, LFP recording was performed from A1 cortex of awake, head-restrained mice, as previously described [2]. The mice were briefly anesthetized with 1% isoflurane to hold the animal head fixed to the stereotaxic instrument using the headpost, and the body was covered with adhesive paper tape to limit body movements. The micro-array electrodes were directly connected, via an EIB-36-Micro headstage pre-amplifier, to a Digital Lynx 4SX system (Neuralynx Inc.), where LFP signals were filtered (bandwidth from 0.1 to 475 Hz), digitized, and acquired at a sampling rate of 1.56 kHz per channel. Thirty minutes after the cessation of anesthesia, LFPs were recorded from A1 cortex of awake, head-restrained mice in a custom-made auditory isolation chamber (background sound level, 40 dB SPL). 500-ms long click trains consisting of 80 dB white-noise pulses presented at 40 Hz [40-Hz auditory steady-state response (ASSR) stimuli] were applied 50 times with an inter-stimulus interval of 20 s. Auditory click stimuli, consisting of white noise pulses (1ms, duration; 80 dB, SPL),

were generated in Labview (National Instruments Inc.), and presented using a speaker with a 35 Hz–20 kHz frequency response (Z3, Logitech Inc.) placed 30 cm above the mouse head.

Each animal was injected intraperitoneally with four different GSK3 inhibitors or vehicle (saline), every third day for each, to have the optimum effect of their action.

Neuralynx LFP files were first converted to Spike2 format to inspect the raw data. Only the channel data in which the amplitude of initial N1 potential evoked by 40 Hz click train was more than 0.1 mV (~4 times the standard deviation), were used for subsequent analyses. Next, LFP voltage values in the Neuralynx files were converted to Matlab (Mathworks) files, and these values were normalized to the z-scores by subtracting the mean and dividing by the standard deviation of the LFP voltages during entire recording epoch (~20 min). The Matlab files with the z-scores were then converted to NeuroExplorer (Nex Technologies) files to calculate the power.

ASSR analyses

The oscillatory components of ASSRs were quantified through time-frequency decomposition of ASSR single trial or averaged data, and analyzed as total power, evoked power, and phase-locking factor (PLF) [3][4]. For total ASSR power, single-trial z-score normalized LFP during the last 200-ms of each ASSR period (500-ms) was analyzed with a fast Fourier transform (FFT) algorithm in the range of 0–100 Hz using 256 frequency bins and averaged the resulting 50 spectra as absolute total ASSR power. Relative (baseline-corrected) total power amplitudes at 35-44 Hz were calculated by subtracting a spontaneous power (35-44 Hz) during inter-stimulus intervals (ISIs) from the absolute total ASSR power (35-44 Hz). The ISI spontaneous power magnitudes were measured as *z*-score normalized LFP from the 200-ms inter-stimulus segment at the mid-time point of ISIs located at 10 s prior to each ASSR click-train onset. For evoked ASSR power, baseline-corrected LFPs, after subtraction of a spontaneous LFP amplitude (200-ms) at the middle of preceding ISI from the z-score normalized evoked LFP amplitude during the last 200-ms of each ASSR period (500-ms), was averaged 50 times, and then analyzed with FFT algorithm in the range of 0–100 Hz using 256 frequency bins. To calculate the consistency in signal phase across single trials, phase locking to click-trains was performed in a frequency range 0-100 Hz with a 60 % overlapping window after applying Hanning tapering of normalized LFP data, which was further analyzed with FFT algorithm. For baseline LFP power at 35-44 Hz during a pre-stimulus period, z-score normalized LFP data during the last 10 s (200-ms bin x 50) prior to the first click-train administration were analyzed with FFT algorithm in the range of 0-100 Hz using 256 frequency bins.

In vivo multi-tetrode recording

In vivo tetrode recording was performed as described [5] with modification. A microarray carrying six tetrodes with an inter-electrode separation of 100 μm was implanted into superficial somatosensory cortex of the right hemisphere (2 mm lateral and 1 mm posterior from bregma) and two stainless steel screws in the frontal cortex which served as ground and reference electrodes. The impedance of each electrode was between 0.2 and 0.3 MΩ. A few days after surgery, mice were connected to an EIB-36 headstage pre-amplifier of a Digital Lynx 4SX system (Neuralynx). The microarray with six tetrodes was slowly lowered over several days using the custom-made microdrive until several multi-unit activities were detected. Multi-unit activities and LFPs were monitored in a low-walled linear track (77 × 7 cm) from the *Grin1* mutant mice, flox control mice, *GSK3A* knockdown mice and *GSK3B* knockdown mice on the *Grin1* mutant background. Multi-unit signals were filtered (bandwidth from 600 to 6 kHz), digitized, and acquired at a sampling rate of 32 kHz per channel and LFP signals were filtered (bandwidth from 0.1 to 475 Hz), digitized, and acquired at a sampling rate of 1.56 kHz per channel. In case of drug experiment, *in vivo* multi-unit activities were recorded from the *Grin1* mutant mice one hour after administration of TDZD-8 or SB216763 following the multi-unit recording without GSK inhibitor. Spike sorting was performed offline with SpikeSort 3D using a combination of KlustarKwik (Neuralynx) and manual procedures. Action potentials (APs) were assigned to individual neurons on the basis of the relative amplitudes across the four recording wires of a tetrode; interspike-interval distribution and auto-correlogram were used to identify the AP that occurred in a refractory period. To be included for analysis, isolated neurons had to fire a minimum of 0.05 Hz during the run session and have less than 0.5% of the neuron spikes fall in a 2-ms refractory period. Putative pyramidal neurons were defined by relatively broad spike waveforms (peak to trough >380 μs) and negative curvilinear after-hyperpolarization. Putative interneurons were defined by

relatively narrow waveforms (peak to trough <380 μs) with positive curvilinear shapes and were not included in the present analysis.

To analyze synchronous spike firing of neighboring pyramidal neurons, we calculated cross-correlogram for spiking from pairs of neurons recorded in the same tetrode with a 5-ms bin width and a time lag of ± 1 s (Neuroexplorer, Nex Technologies). For each pair of neurons, the cross-correlation was considered to be significant if the peak/trough was within ± 100 ms of the zero-lag point and greater than the 99% confidence interval. The magnitude of the cross-correlations was measured as the peak (or absolute value of significant trough in the case of anti-correlated pairs) minus chance (assuming independent neuron firing with a Poisson distribution) to correct for differences in mean firing rates across populations.

Ex vivo multiple patch clamp recording

Coronal brain slices containing A1 cortex were prepared from mice (both sex) at 4-6 weeks of age. Mice were deeply anaesthetized with isoflurane and decapitated. Brain was removed within 30 s and immediately placed in ice cooled slicing aCSF, containing (in mM) 234 Sucrose, 2.5 KCl, 25 $NaHCO_3$, 1.25 $NaHPO_4$, 25 Glucose, 0.5 $CaCl_2$, 7 $MgCl_2$, 3 Myo-inositol, 2 Na-pyruvate, and 0.4 ascorbic acid; the pH was maintained to 7.4 under continuous bubbling with carbogen gas (95% O_2/5% CO_2). Three hundred μm-thick coronal slices were prepared using Compresstome vibrating microtome (VF-310-0Z, Precisionary Instruments, USA). Thereafter, slices were incubated at 35 ºC in recording aCSF containing (in mM) 125 NaCl, 2.5KCl, 25 $NaHCO_3$, 1.25 $NaHPO_4$, 25 Glucose, 2 $CaCl_2$, 1 $MgCl_2$, 3 Myo-inositol, 2 Na-pyruvate, and 0.4 ascorbic acid, pH 7.4, for 30 min and then kept at room temperature until used for the experiments.

Simultaneous multiple whole-cell voltage-clamp recordings were performed from layer 2/3 pyramidal neurons. Brain slices were put in a recording chamber continuously perfused with aCSF at flow rate of 2-3 ml per min. Patch pipettes were pulled with Flaming Brown micropipette puller (P97, Sutter Instrument Co. USA) and had resistance of 3-5 MΩ when filled with intracellular buffer, in mM: 140 KCl, 10 HEPES, 4 Mg-ATP, 0.4 Na-GTP, 10 Na-Phosphocreatine and 5 QX-314, pH adjusted to 7.3 with KOH. Simultaneous multiple whole cell voltage clamp recordings were performed on layer 2/3 pyramidal neurons holding the membrane potential at -70 mV. Series resistance was in all cases <20 MOhm and not compensated. All recordings were done at 30-32 ºC using EPC10 USB Quadro, HEKA Instruments Inc. USA. Inhibitory postsynaptic currents (IPSCs) were isolated by adding 50 μM D-2-Amino-5-phosphopentanoic acid (D-AP5) and 20 μM 6-Cyano-7-nitroquinoxaline-2,3-dione (CNQX) to the recording aCSF. Spontaneous IPSCs were detected and analyzed using custom written Igor procedures by setting event detection threshold at 5 pA. Time stamps, of IPSC activation, generated by Igor analysis were imported to Microsoft Excel sheet and synchronous events between pairs of neurons were detected by custom written Excel macro by defining the spontaneous IPSCs coinciding within ± 10 ms of signal window.

Y-maze spontaneous alternation task

A Y maze with three identical arms of transparent Plexiglas (40 × 4.5 × 12 cm) 120° apart was placed in the center of a diffusely illuminated room (30 lx) with clues located in the periphery of the room to allow visual orientation. In case of drug treatment, TDZD-8, BRD3731 or BRD0705 was administrated to the naïve mutant mice and saline was *i.p.* administrated to their naïve control mice one hr before Y maze spontaneous alternation task. Each mouse was placed at the end of one arm facing the center and allowed to freely explore the apparatus with the experimenter out of sight. All sessions were video recorded through a camera mounted above the maze. Entries into each arm were scored for eight min starting at the first entry. Alternation behavior was defined as consecutive entries into each of the three arms without repetition (that is, ABC, BCA…). We defined the percentage of spontaneous alternation as the actual alternations divided by the possible alternation (total arm entries − 2) × 100. Total entries were scored as an index of ambulatory activity in the Y maze. Mice with scores below 12 were excluded. All experiments were conducted during the initial dark phase (6:00 p.m. to 9:00 p.m.) to maximize exploratory behavior.

Prepulse Inhibition (PPI)

TDZD-8, BRD3731 or BRD0705 was administrated to the mutant mice and saline was administrated to their control mice by *i.p.* injection one hour before PPI. Each session consisted of 42 trials, following a 5-min acclimation period. Six different trial types were presented: the no-stimulus trials, trials with the acoustic startle stimulus (40 ms, 120 dB) alone, and trials in which a prepulse

stimulus (20-ms white noise, either 72, 74, 78 or 84 dB) applied 100 ms before the onset of startle stimulus. The different trial types were presented in blocks of six, in randomized order in each block, with an average intertrial interval of 15 s (range, 10–20 s). Each session was initiated with a 5-min acclimation period of background noise (70 dB) followed by seven successive 120-dB habituation tones (40 ms long, intertrial interval of 15 s). These trials were not included in the analysis. Measures were taken of the startle amplitude for each trial, defined as the peak response during a 65-ms sampling window that began with the onset of the startle stimulus. An overall analysis was performed for each subject's data for levels of PPI at each prepulse sound level (calculated as 100 − (response amplitude for prepulse stimulus and startle stimulus together/response amplitude for startle stimulus alone) × 100).

Supplementary Result and Discussion

"Evoked power", "Total power" and "PLF" are three major time-frequency measures of event-related oscillatory power [3][4]. Evoked power is the result from pure-phase resetting of ongoing oscillations by a stimulus. On the other hand, total power is the result from the change in power relative to the pre-stimulus baseline [6]. Accordingly, there is an argument that total gamma power may contain not only evoked power component but also induced power component, which could dilute the effect of the event related activities [7]. In our previous study to assess the ASSR deficits in the *Grin1* mutant mice, we measured "total ASSR power" and PLF, both of which were robustly impaired in the mutant mice, although we referred the total power as "evoked power" in that report [2]. In the present study, we calculated total power and evoked power, as well as phase locking, to quantify 40-Hz ASSR deficits and the effect of GSK3β inhibition in the *Grin1* mutant mice. The evoked power data and PLF data are presented in the main text, and total power data is presented in the *Supplementary Information*. We found that mutant ASSR deficits and the rescue effects by GSK3β inhibition are consistently detected across all three time-frequency measures, suggesting the robustness of the mutant phenotypes and its rescue by GSK3β inhibition, and support for the use of these three 40-Hz ASSR measures. Notably, among power analyses with the floxed-control ASSR data, the evoked gamma power amplitudes were larger than total gamma power amplitudes (Supplementary Fig. S3D), which may make the evoked power analysis more sensitive compared to the total power analysis. However, limited sample size precluded drawing such a conclusion.

Supplementary References

1. Santarelli R, Carraro L, Conti G, Capello M, Plourde G, Arslan E. Effects of isoflurane on auditory middle latency (MLRs) and steady-state (SSRs) responses recorded from the temporal cortex of the rat. Brain Res. 2003;973:240–251.
2. Nakao K, Nakazawa K. Brain state-dependent abnormal LFP activity in the auditory cortex of a schizophrenia mouse model. Front Neurosci. 2014;8:168.
3. Roach BJ, D'Souza DC, Ford JM, Mathalon DH. Test-retest reliability of time-frequency measures of auditory steady-state responses in patients with schizophrenia and healthy controls. NeuroImage Clin. 2019;23:101878.
4. Javitt DC, Siegel SJ, Spencer KM, Mathalon DH, Hong LE, Martinez A, et al. A roadmap for development of neuro-oscillations as translational biomarkers for treatment development in neuropsychopharmacology. Neuropsychopharmacology. 2020;45:1411–1422.
5. Belforte JE, Zsiros V, Sklar ER, Jiang Z, Yu G, Li Y, et al. Postnatal NMDA receptor ablation in corticolimbic interneurons confers schizophrenia-like phenotypes. Nat Neurosci. 2010;13:76–83.
6. Mathalon DH, Sohal VS. Neural Oscillations and Synchrony in Brain Dysfunction and Neuropsychiatric Disorders. JAMA Psychiatry. 2015;72:840.
7. David O, Kilner JM, Friston KJ. Mechanisms of evoked and induced responses in MEG/EEG. Neuroimage. 2006;31:1580–1591.
8. Patel S, Doble BW, MacAulay K, Sinclair EM, Drucker DJ, Woodgett JR. Tissue-specific role of glycogen synthase kinase 3 in glucose homeostasis and insulin action. Mol Cell Biol. 2008;28:6314–6328.

Supplementary Figures

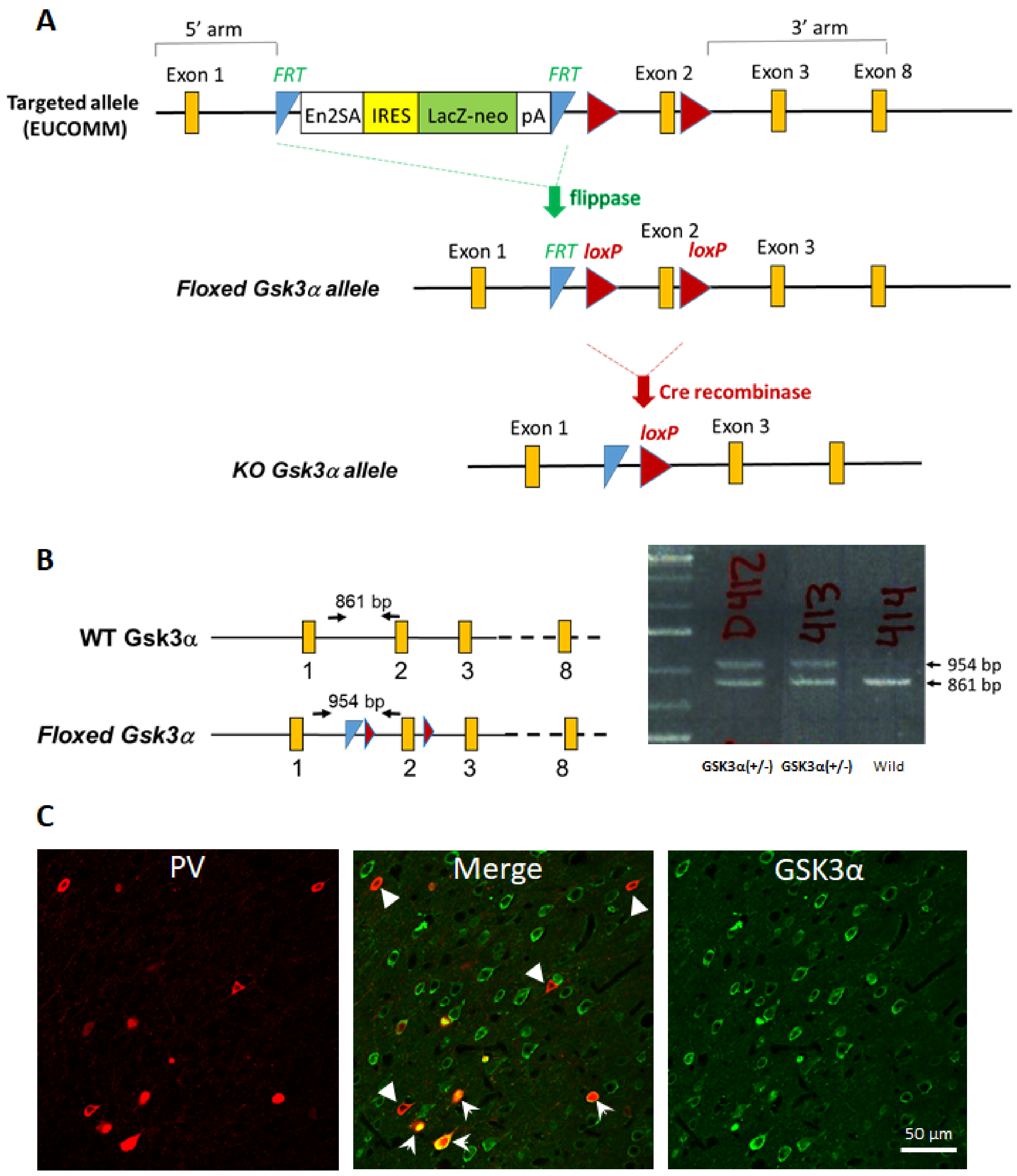

Figure S1. **Generation of *GSK3A* floxed mice.**
(**A**) The ES cell (#HEPD0506_1_B11) containing an Frt flanked IRES:LacZ-neo trapping cassette and two loxP sequences flanking exon 2 of GSK3A allele was purchased from European Mouse Mutant Archive (EMMA), and conditional knockout mice were generated at the Transgenic & Genetically Engineered Models Core Facility at the University of Alabama at Birmingham (UAB). After crossing to a ROSA26-flippase (FLPe) knock-in mice (Jax; #003946), the removal of Frt flanked cassette was confirmed by the PCR.

(**B**) Two oligonucleotide primers are used to distinguish the wild-type (WT) allele and the floxed allele of *GSK3A*; *GSK3A*-con-F: 5'- CCC CCT TGC AAA TTG CCT TTG TTG G -3', *GSK3A*-con-R2: 5'-GGT CAC CTT CCC GCT GTC ACC -3'. Right panel in (B) is the example results of *GSK3A* PCR genotyping.
(**C**) Cre recombination and subsequent removal of exon 2 of GSK3α allele in GABA neurons was confirmed by brain immunocytochemistry using anti-GSK3α antibody (G08-63R-25, SignalChem, USA) in the Ppp1r2cre/homozygously floxed-*GSK3A* mouse line. Representative confocal photomicrographs from coronal section double-immunostained with anti-PV (left), anti-GSK3α (right) and merge (middle). Large arrowheads indicate no GSK3α protein in PV neurons by cre-recombination, verifying the conditional *GSK3A* knockout. Small arrow heads represent GSK3α immunoreactivity presumably in Cre-negative PV neurons. Note that about 83.5% of cortical PV neurons are positive for Cre in Ppp1r2cre line (see Fig. S10 in [5]). Generation of *GSK3B* floxed mice was described by [8].

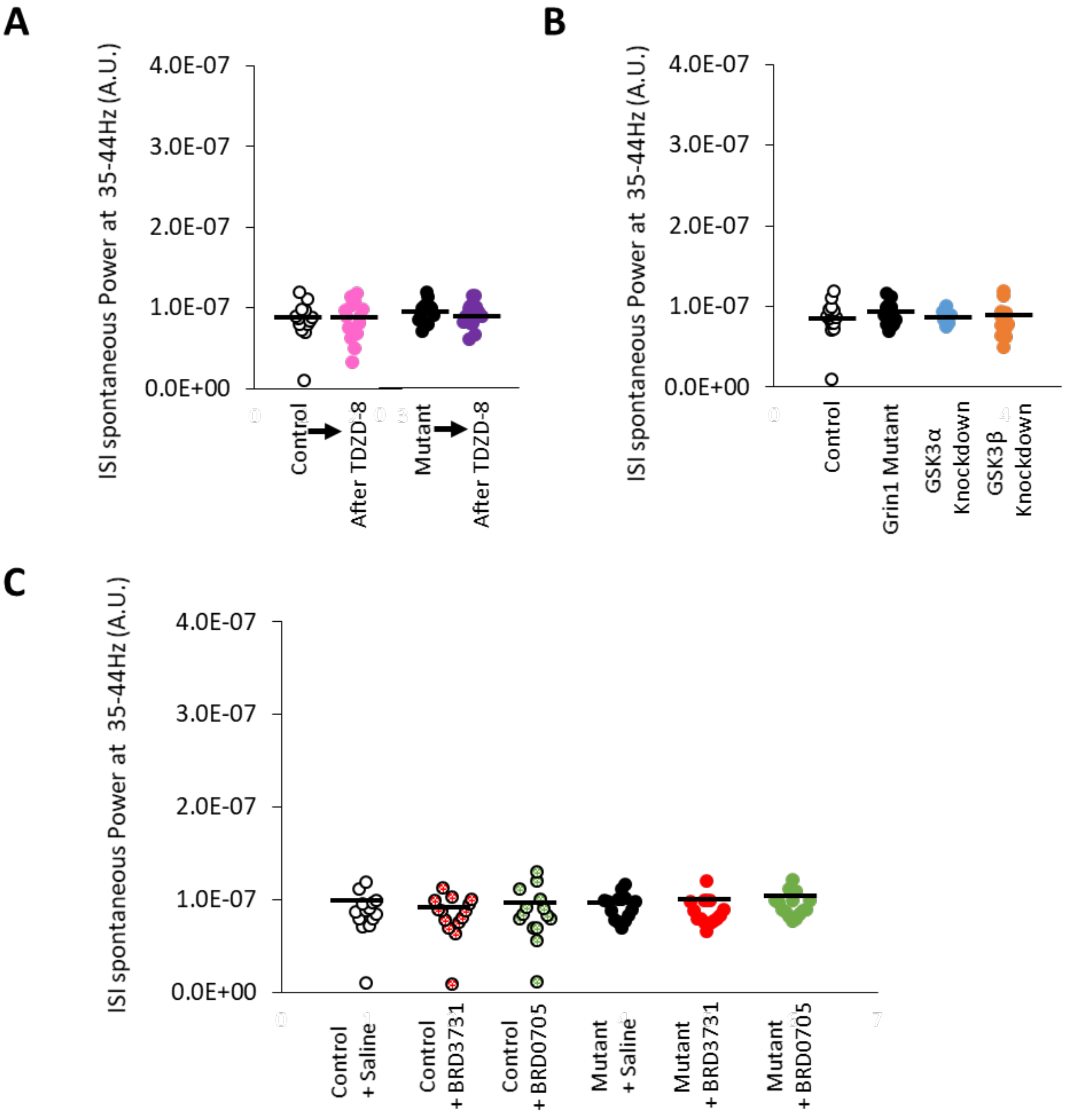

Figure S2. No effect of GSK3 inhibition on the magnitude of spontaneous LFP power during inter-stimulus intervals (ISIs) of periodic click-train stimuli.
(**A**) TDZD-8 did not alter the spontaneous LFP powers at 35-44 Hz during inter-stimulus interval (ISI) of tone stimuli in control mice (14 channels) and *Girn1* mutant mice (15 channels). (**B**) ISI spontaneous gamma power (z-score) was unaffected by GSK*3A* and *GSK3B* knockdown (14 channels from floxed-control mice, 15 channels from *Grin1* mutant mice, 6 channels from three *GSK3A* knockdown mice on the mutant background and 14 channels from *GSK3B* knockdown mice on the mutant background). (**C**) Administration of either BRD3731 or BRD0705 showed no impact on ISI spontaneous power at 35-44 Hz (Mutant with Saline vs. Mutant with BRD3731, p = 0.94, Mutant with Saline vs. Mutant with BRD0705, p =1.00, two-way ANOVA with Tukey-Kramer *post hoc* test).

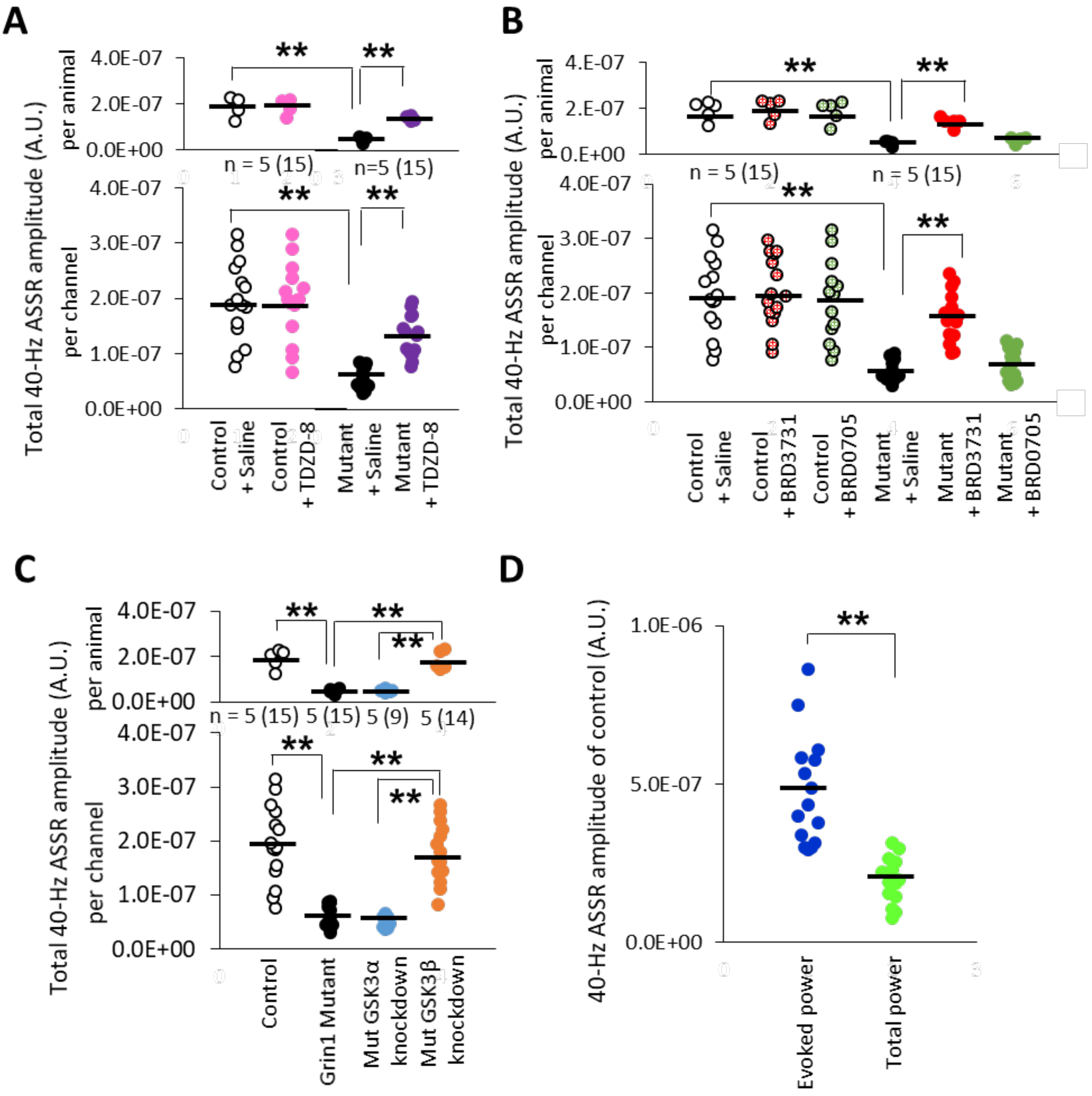

Figure. S3 GSK3β inhibitor alleviated total gamma oscillation deficits in *Grin1* mutant mice.
(**A**) Total 40-Hz ASSR amplitude was evaluated before and after i.p. injection of TDZD-8 (non-selective GSK3 inhibitor) in control (15 channels) and mutant mice (15 channels) per animal (top) and per channel (bottom) [before TDZD-8 treatment, controls vs mutants, $F(1,27) = 40.5$, Drug × Genotype, $p < 0.001$ (per channel), $F(1,7) = 65.6$, $p < 0.001$ (per animal), two-way Repeated Measures ANOVA with Tukey-Kramer post hoc test]. TDZD-8 normalized total ASSR power at 40Hz in Grin1 mutant mice [($p < 0.001$ (per channel), $p < 0.001$ (per animal), two-way Repeated Measures ANOVA with Tukey-Kramer post hoc test), but not in the control mice [$p = 1.00$ (per channel), $p = 0.95$ (per animal)]. (**B**) Total 40-Hz ASSR before and after *i.p.* injection of either BRD3731 or BRD0705 in floxed-control (15 channels) and mutant mice (15 channels). BRD3731, but not BRD0705, alleviated ASSR power deficits at 40Hz in *Grin1* mutant mice by per-animal and per-channel analyses [15 channels before and after BRD3731, $F(2,81) = 4.67$, drug × genotype, $p < 0.001$ (per channel), $F(2,21) = 3.69$, $p < 0.01$ (per animal); 15 channels before and after BRD0705, $p = 0.998$ (per channel), $p = 0.99$ (per animal), two-

way ANOVA with Tukey-Kramer *post hoc* test]. Neither BRD3731 nor BRD0705 affected the total-gamma oscillation in floxed-control mice (before and after BRD3731, $p = 0.99$; before and after BRD0705, $p = 1$, two-way ANOVA with Tukey-Kramer *post hoc* test). (**C**) Total 40-Hz ASSR amplitudes were evaluated in floxed-control mice (15 channels), *Grin1* mutant mice (15 channels), GSK3A knockdown mice on *Grin1* mutant background and GSK3B knockdown mice on the mutant background (14 channels). GSK3B knockdown in Grin1 mutant mice, but not GSK3A knockdown, normalized 40-Hz ASSR power assessed both by per-animal (top) and per-channel (bottom) designs (GSK3B knockdown vs original *Grin1* mutant mice, $F(3, 45) = 26.9$, $p < 0.001$ (per channel), $F(3, 13) = 24.8$, $p < 0.001$ (per animal), one-way ANOVA with Tukey-Kramer *post hoc* test). (**D**) Evoked 40-Hz ASSR amplitudes in control mice were bigger than total 40-Hz ASSR amplitudes. $^{**}p < 0.001$, Student's *t*-test.

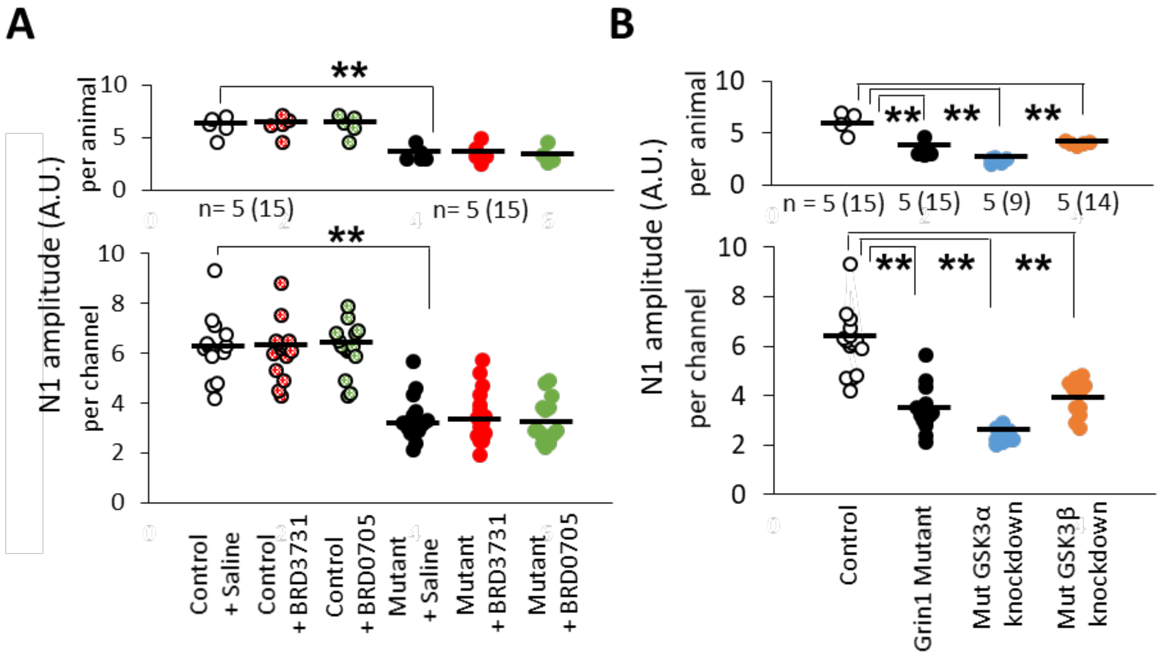

Figure. S4 *GSK3B* inhibitor did not alter the N1 amplitudes in *Grin1* mutant mice.
(**A**) BRD3731 or BRD0705 did not alter the N1 amplitudes in *Grin1* mutant mice by both per-animal and per-channel analyses (15 electrode channels before and after BRD3731, p = 0.99 (per channel), p = 0.99 (per animal); 15 electrode channels before and after BRD0705, p = 1.00 (per channel), p = 1.00 (per animal), two-way ANOVA with Tukey-Kramer *post hoc* test). (**B**) GSK3B knockdown did not alter the N1 amplitudes evoked by click-train stimuli ($F(3, 45) = 30.6$, $p = 0.35$ (per channel), $F(3,13) = 34.1$, $p = 0.35$ (per animal), one-way ANOVA with Tukey-Kramer *post hoc* test).

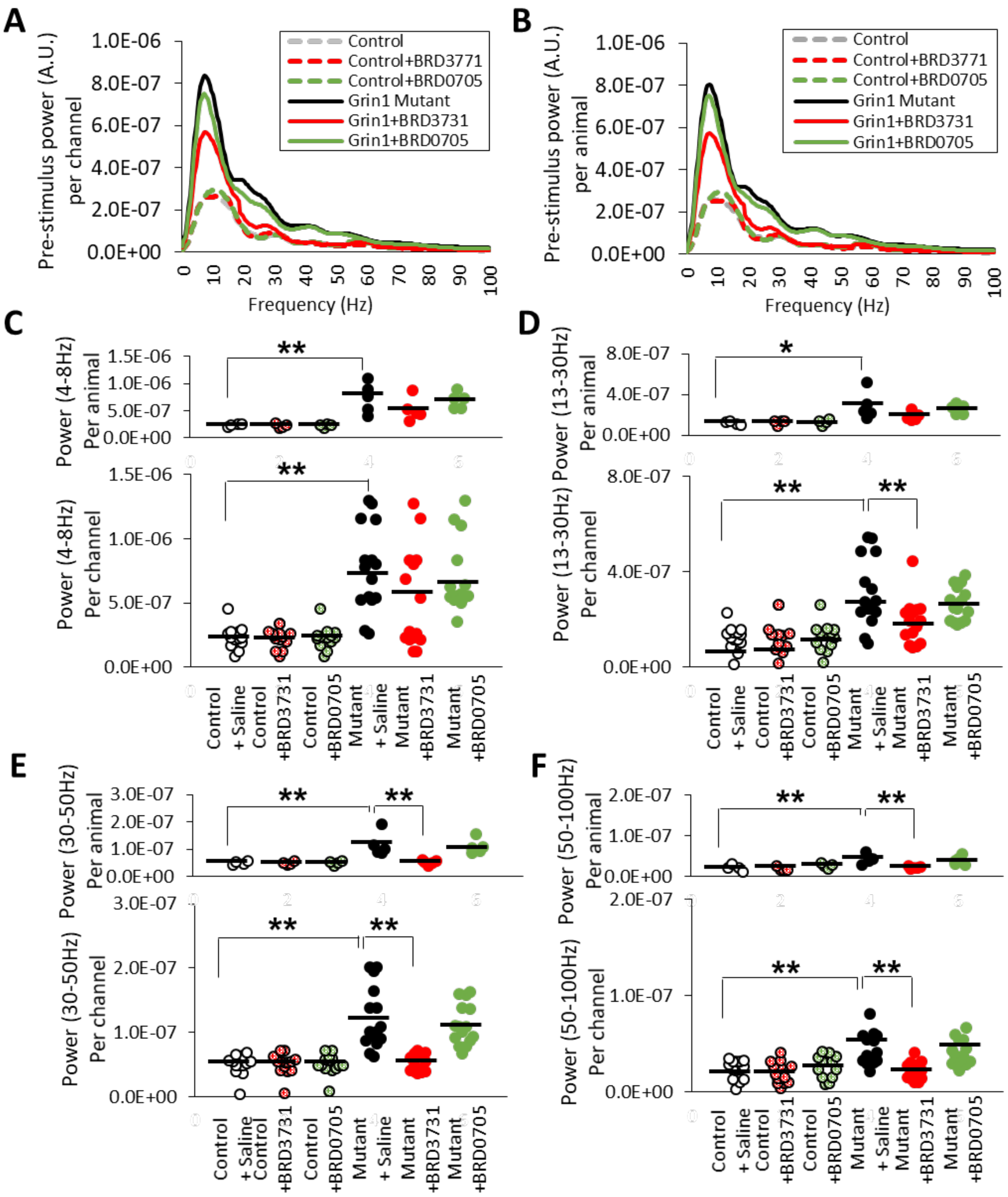

Fig. S5. Paralog-selective GSK3β inhibitor (BRD3731), but not GSK3α inhibitor (BRD0705), alleviated elevated baseline gamma in *Grin1* mutant mice.
(A) (B) *Z*-score normalized spectral density power during the pre-stimulus period (10 s) from control (14 channels) and mutant mice (15 channels) per channel (A) and per animal (B) before and after *i.p.* injection of BRD3731 and BRD0705. **(C)** Averaged baseline LFP powers at theta (4-8 Hz) frequency per channel (bottom) and per animal (top). BRD3731 and BRD0705 had no effect of theta power. (**D**) BRD3731 rescued baseline beta (13-30 Hz) power in per-channel analysis (bottom), but not per-animal analysis. (**E, F**) Low gamma (30–50 Hz) and high gamma (50–100 Hz) frequency baseline power increase were reversed by BRD3731, but not BRD0705. ***p* < 0.01 and **p* < 0.05, two-way ANOVA with Tukey-Kramer *post hoc* test.

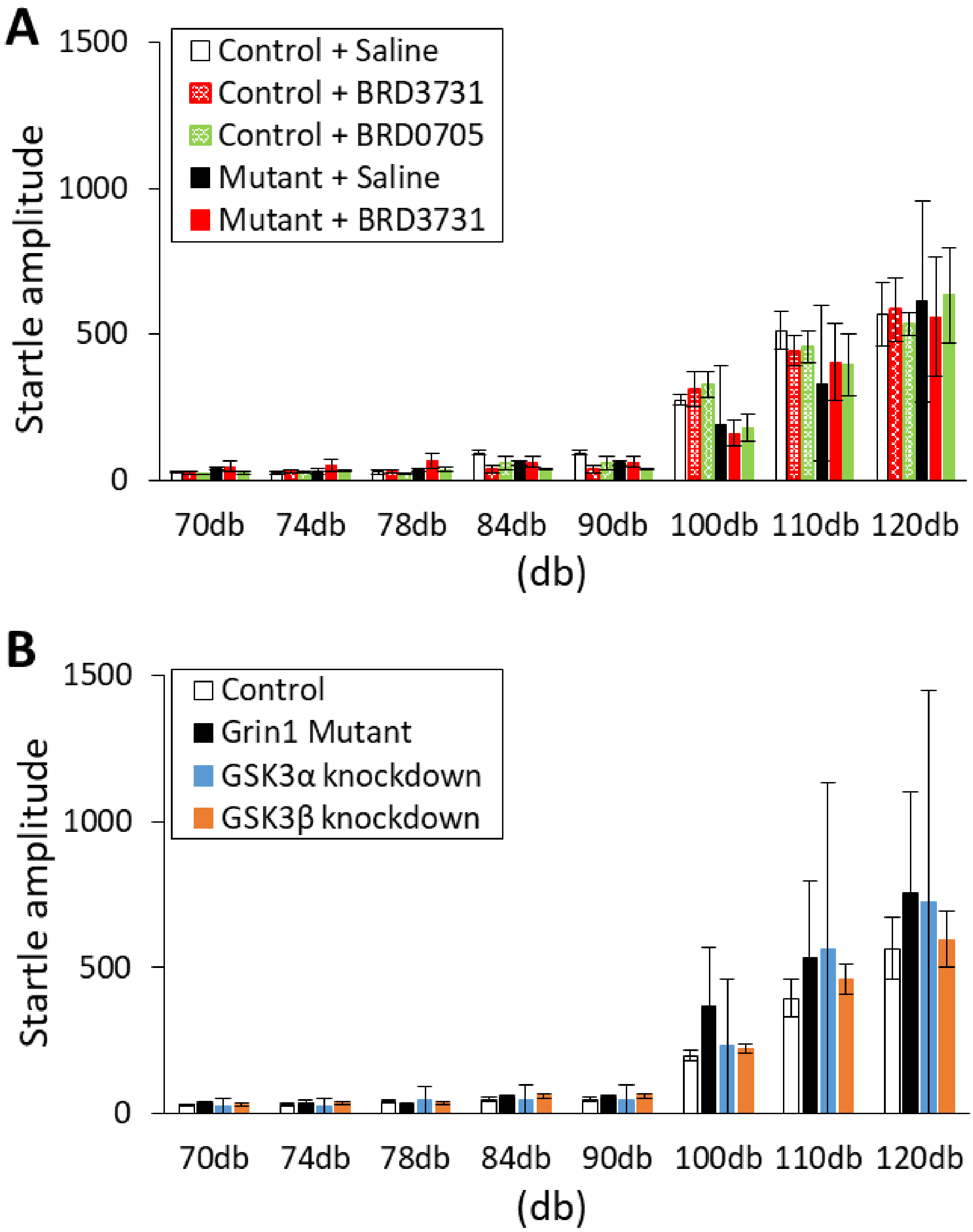

Figure S6. GSK3β inhibition showed no effect on the acoustic startle.
(**A**) Systemic treatment of paralog-selective GSK3 inhibitor, BRD3731 and BRD0705, showed no impact on the startle amplitudes (n =11 for BRD3731 vs n =7 for original *Grin1* mutants, p =1.00, n =8 for BRD0705 vs n =7 for original *Grin1* mutants, p =1.00, Repeated Measures ANOVA Tukey-Kramer *post hoc* test). (**B**) Neither GABAergic neuron-selective *GSK3A* nor *GSK3B* knockdown altered the startle (n = 5 for *GSK3A* knockdown vs n = 7 for original *Grin1* mutants, p =1.00, n = 8 for *GSK3B* knockdown vs n = 7 for original *Grin1* mutants, p =1.00, Repeated Measures ANOVA Tukey-Kramer *post hoc* test).
